# Self-Healing 3D-Printed Polyurethane Nanocomposites Based on Graphene

**DOI:** 10.3390/mi16080889

**Published:** 2025-07-30

**Authors:** Justyna Gołąbek, Natalia Sulewska, Michał Strankowski

**Affiliations:** 1 Polymer Technology Department, Chemical Faculty, Gdansk University of Technology, 80233 Gdansk, Poland; justyna.golabek@pg.edu.pl (J.G.);; 2 Beckman Institute for Advanced Science and Technology, University of Illinois at Urbana-Champaign, Urbana, IL 61801, USA

**Keywords:** polyurethanes, graphene, nanocomposites, hydrogen bonds, Ureido-pyrimidinone, self-healing materials, thermal analysis, 3D printing

## Abstract

This study explores the self-healing properties of polyurethane nanocomposites enhanced by multiple hydrogen bonds from ureido-pyrimidinone and the incorporation of 1–3 wt.% graphene nanoparticles, based on polyol α,ω-dihydroxy[oligo(butylene-ethylene adipate)]diol, which, according to our knowledge, has not been previously used in such systems. These new materials were synthesized via a two-step process and characterized by their thermal, mechanical, chemical, and self-healing properties. The mechanical analysis revealed that all nanocomposites exhibited high self-healing efficiencies (88–91%). The PU containing 2% graphene stands out as it exhibits the highest initial mechanical strength of ~5 MPa compared to approximately 2MP for a pristine PU while maintaining excellent self-healing efficiency (88%). A cut on the PU nanocomposite with 2% graphene can be completely healed after being heated at 80 °C for 1 h, which shows that it has a fast recovery time. Moreover, 3D printing was also successfully used to assess their processability and its effect on self-healing behavior. Three-dimensional printing did not negatively affect the material regeneration properties; thus, the material can be used in a variety of applications as expected in terms of dimensions and geometry.

## 1. Introduction

In recent years, there has been an increasing emphasis on the fact that materials must be progressed in accordance with the principles of sustainable development, not forgetting the ecological aspect, since the production of plastics based on components of petrochemical origin has a huge, often negative impact not only on the environment but also on human health, and at the same time, all new materials are increasingly demanding not only in terms of their manufacture but also their properties [1]. Modern smart materials are required to have improved mechanical properties, durability, and high functionality to ensure the highest possible performance in applications such as “smart” wearable devices [2,3], energy storage devices [4,5], next-generation sensors and actuators [6,7], etc. One polymer of considerable interest to meet the above-mentioned requirements is polyurethane (PU). Specifically, thermoplastic polyurethane (TPU) is valued because it exhibits thermoplastic properties that allow it to be reprocessed at elevated temperatures using various manufacturing methods such as extrusion, injection molding or compression molding, solution coatings, and nowadays even 3D printing [8]. TPU is a linear multi-block copolymer consisting of alternating soft and hard segments. These segments are formed by the addition polymerization of isocyanates and chain extenders with polyols. Isocyanates and chain extenders are assembled by hydrogen bonds of urethane groups to form a hard phase, which is dispersed in a soft phase containing flexible polymer chains. This structure results in microphase separation, which endows PUs with good mechanical properties and high elasticity and resilience. Moreover, by using different components and changing the ratios between hard and soft segments, we can control the structure of the separated phases to a certain extent, thus adapting the properties of PU to our needs [9]. However, like any other material, PU materials are susceptible to mechanical damage and microcracking, which significantly reduces their performance, leading to a significantly reduced service life [10].

Thus, for many years, multitudes of researchers have been working on ways to extend the lifespan of materials by introducing self-healing functions via an extrinsic or intrinsic mechanism. In the extrinsic mechanism, a capsule or vascular system filled with a healing agent is embedded in a polymer matrix. When the material is mechanically damaged, the capsule/vascular system ruptures and the healing agent is released to fill the cracks. Extrinsic self-healing is particularly effective in restoring a large part of the material. The biggest disadvantage is the one-time-use nature of the process when the structure is ruptured due to the limited supply of capsules [8,11]. Recently, of particular interest in the context of polyurethanes are so-called supramolecular polyurethanes [12] based on reversible interactions introduced as dynamic covalent bonds or non-covalent bonds. Intrinsic self-healing is based on material-specific properties, i.e., reversible cracking and recombination of chemical bonds to repair damaged areas [13]. Intrinsic self-healing based on disulfide bonds [14], Diels–Alder bonds [15], imine bonds [16] and boronic ester bonds [17], hydrogen bonds [18], metal coordination interactions [19], and host–guest interactions [20] enables multiple healing cycles.

Despite many efforts, it has been found that the introduction of a single type of interaction is not effective, so one of the main challenges facing self-regenerating materials is determining how to prevent the loss of strength and durability while maintaining regenerative ability. Yang et al. [21] prepared polyurethane elastomers based on non-covalent quadruple hydrogen bonding achieved by using polyether amines of two different molecular weights. They found that by changing the ratio between the two polyether amines, the density of hydrogen bonds between molecular chains could be affected, thus giving the elastomers self-healing properties. However, increasing the ratio to a certain critical point leads to an increase in hydrogen bond density and an increase in the relaxation time of the molecular chains to the point where a decrease in self-healing performance is observed. None of the healed elastomers based on two types of diamines exceeded the mechanical strength of the original sample, reaching only 5 MPa and a self-healing efficiency of up to 82% after 10 h of regeneration at room temperature, which does not give satisfactory results. Nevejans et al. [22] synthesized a series of water-based poly(urethane-urea)s (PUUs) containing covalent disulfide bonds or C-C bonds, both linear (L-S and L-C) and cross-linked (X-S and X-C). The results showed that PUUs with disulfide bonds achieved only 16.3 MPa tensile strength and 315% strain at break compared to 28.8 MPa and 395% for C-C based materials, respectively. During the scratch gap closure experiment, the scratching of the L-S sample was almost invisible after 2 h of healing at 80 °C compared to the scratching of the L-C sample that was still present, with similar conclusions being drawn for cross-linked materials. However, it should be noted that although the regeneration of disulfide bond-based materials is higher compared to C-C-based PUUs, the total crack propagation energy obtained during fracture mechanics experiments was higher for C-C samples, implying that they have higher mechanical strength. In conclusion, dynamic bonds provide the ability to self-heal by accelerating healing kinetics; however, they are strongly dependent on mechanical properties as materials are more prone to fracture at high stress, clearly demonstrating the importance of material design.

One strategy to overcome these challenges is to use both covalent and non-covalent interactions to achieve a synergistic effect that can lead to improved properties [23,24,25,26]. Li et al. [27] synthesized a supramolecular polyurethane elastomer containing multiple hydrogen bonds (Urea-4 [1H]-pyrimidinone (UPy)) and coordination bonds (Zn^2+^) showing a high tensile strength of 30 MPa, a repair efficiency of 87% (12 h at 45 °C), a high elongation at break of about 925%, and high transparency reaching 90% transmittance. Moreover, the synergistic effect between non-covalent and covalent bonds ensured good recyclability; mechanical properties remained high even after 14 recycling/reshaping cycles. The introduction of multiple hydrogen bonds enabled reorganization in a dynamic manner at the rupture site with the simultaneous restoration of coordination bonds, which affected the movement of polymer chain segments, leading to successful healing of the polyurethane film. In another study [28], the polyurea material was enhanced by dynamic imine and hydrogen bonds, where the self-healing properties were induced by the presence of water, resulting in a tensile strength of 41 MPa, a toughness of 127 MJ/m^3^, and a strain at break of 823%. Very high healing efficiency was observed after just 2 h at 60 °C, reaching 92%, and also achieving 93% at room temperature after 72 h. As in the previously cited work, the synergistic effect also influenced the high processability of the material; after three cycles of cutting/hot-pressing, there were no significant changes in the FTIR spectra, indicating that both imine and hydrogen bonds were still present in the structure of the material, enabling multiple self-healing processes. Excellent results were also obtained by incorporating quadruple hydrogen bonds and Diels–Alder bonds into the polyurethane system, showing excellent mechanical strength of ~52 MPa, toughness of 166.7 MJ/m^3^, stretchability of up to 930%, and very high healing efficiency of 91%. The polyurethane sample was evaluated also as a substrate for self-healing conductive devices. The conductive device (polyurethane film coated with silver glue) was connected in circuits with a light bulb. When the material was cut, the bulb went out. After a period of healing, the path was restored, and the bulb was lit again [29].

Despite many advantages, even this approach has shown some limitations in terms of mechanical strength and self-healing performance. Consequently, another path has also been taken, which involves the addition of nanoparticles to develop nanocomposites with noticeable results in improving the chemical and physical properties of modern materials. Tian et al. [30] improved the mechanical properties of polyurethane coatings containing disulfide bonds by adding imidazolium-modified carboxylated graphene (FCGO). The tensile stress improved by 40% compared to the pristine material, reaching almost 21 MPa, while maintaining healing efficiency close to 80% after 48 h at 60 °C. Yan et al. [23] prepared self-healing waterborne polyurethane nanocomposite systems; aromatic and aliphatic disulfide bonds were responsible for the formation of a dynamic reversible crosslinking network during multiple cycles of rupture and recombination, while UPy motifs grafted onto cellulose nanocrystals enhanced the phase separation of hard and soft segments and the bonding interaction at the polyurethane interface. By increasing the UPy-CNC content, hydrogen bond interactions in the polyurethane matrix were enhanced, improving the stiffness and toughness of the composites, which reached 48 MPa and 142 MJ/m^3^, respectively. During healing after cracking at 80 °C for one day, the repair efficiency remained high, reaching more than 82%, 88%, and 77% for crack elongation, tensile stress, and toughness, respectively.

Graphene and its derivatives incorporated into the structure of materials significantly improve mechanical and thermal properties [31]. Luo et al. [32] incorporated graphene oxide into polyurethane to obtain a multifunctional composite with self-healing and shape memory properties. The tensile test results show that the Young’s modulus of the nanocomposites increases from 38.57 MPa for pure polyurethane to 95.36 MPa for the polyurethane composite containing 0.5 wt.% GO. Similarly, the tensile strength improves from 6.28 ± 0.67 MPa to 15.65 ± 1.54 MPa. The presence of oxime–carbamate and hydrogen bonds provides the composite with excellent self-healing properties, achieving a healing efficiency of up to 98.84%. Additionally, the composite exhibits outstanding shape memory behavior with a shape recovery ratio of 88.6% and a shape fixation ratio of 55.2%.

Graphene-based self-healing demonstrates that physical reinforcement with nanofiller, together with a chemical reversible healing mechanism, enables improved mechanical properties, electrical and thermal conductivity, and responsiveness to external stimuli. Currently, the most popular materials in the field of self-repairing polyurethanes modified with graphene, its derivatives, and other nanoparticles are coatings exhibiting barrier properties, i.e., UV shielding [33], anticorrosion protection [34,35], reinforced fabric coating [36], and superhydrophobic coating [37], as evidenced by the large number of scientific articles [38,39,40,41]. Other applications include sensors designed to monitor human movement, heart rate, posture, and health status [42,43,44]. In addition, revolutionary 3D printing technology makes it possible to create complex structures with precisely controlled properties and dimensions [45,46]. Therefore, the combination of 3D printing with polyurethane nanocomposites may define new ways of manufacturing and designing advanced engineering materials in aerospace, automotive applications, electronics, and conductive polymer composites [47,48].

However, most of the cited publications focus on graphene derivatives, such as graphene oxide or reduced graphene oxide, while there is a lack of publications on the effect of graphene addition. Therefore, in the present study, a self-healing polyurethane was synthesized using 4,4′-diphenylmethane diisocyanate (MDI) and α,ω-dihydroxy[oligo(butylene-ethylene adipate)]diol as primary components. To our knowledge, based on current scientific publications, this polyol has not yet been tested in similar self-healing polyurethane systems. To promote supramolecular interactions in the materials, 2-amino-4-hydroxy-6-methylpyrimidine (AHMP), a self-complementary hydrogen bonding motif known as ureido-pyrimidinone (UPy), was used as the copolymerization monomer. At the same time, non-commercial graphene (Gr) obtained in the laboratory was introduced as a physical filler to improve mechanical performance. Moreover, 3D printing was used to design a material with a tailored structure while maintaining appropriate final properties to determine its application potential.

## 2. Materials and Methods

### 2.1. Materials

To obtain materials for research, the synthesis of polyurethanes was carried out using a two-step method. Polyurethane reference material (without the addition of graphene) and polyurethane materials modified with graphene with the addition of 1%, 2%, and 3% by mass, respectively, were obtained. The following raw materials were used for the synthesis of polyurethanes and polyurethane nanocomposites with the addition of graphene: Polyol α,ω-dihydroxy[oligo(butylene-ethylene adipate)]diol (Polios 55/20, Mn = ~2000 g/mol) prod. PURINOVA, Bydgoszcz, Poland; diisocyanate 4,4′-diphenylmethane diisocyanate (MDI, ONGRONAT^®^ 3000) prod. BorsodChem, Kazincbarcika, Hungary; chain extender 1,4-butanediol (BDO, degassed and dehydrated at 80 °C under vacuum for 1 h) + catalyst 1,4-diazabicyclo[2.2.2]octane (DABCO^®^ 33-LV) prod. ThermoFisher Scientific, Waltham, MA, USA and Sigma Aldrich, Burlington, VT, USA, respectively; modifier 2-amino-4-hydroxy-6-methylpyrimidine (AHMP) prod. ThermoFisher Scientific, Waltham, MA, USA; Solvent N,N-dimethylformamide (DMF) prod. Merck, Darmstadt, Germany; and nano-additive in the form of graphene (Gr, previously dried at 80 °C for 4 h), which was developed at the Department of Corrosion and Electrochemistry of the Gdańsk University of Technology (graphene nanofiller was obtained electrochemically in a 0.1 M (NH_4_)_2_SO_4_ solution at 10 V, using 99.9% C graphite electrodes with a 30 mm electrode gap).

### 2.2. Synthesis

A series of polyurethane elastomer materials were prepared using a two-step prepolymer method. In the first step, the polyol was degassed and dehydrated at 70 °C for 2 h under vacuum, followed by the addition of diisocyanate excess. The reaction was carried out at 80 °C at a stirring speed of 150 rpm for 1 h under atmospheric pressure and 1 h under vacuum to prepare a prepolymer with terminal isocyanate groups. After the first step, it was analytically determined that the final content of unreacted NCO groups was 5.84% according to PN-EN 1242, Determination of isocyanate groups content, Warsaw, Poland, 2006.

Part of the resulting prepolymer (60 g) was taken from the reactor system and transferred to a second vessel, after which the DMF solvent was added. After a pronounced change in viscosity, a solution consisting of a low-molecular-weight chain extender along with a catalyst was added. After adding the extender, the speed of the mechanical stirrer increased to 1500 rpm. After 30 s, the polymer was poured onto Teflon plates previously heated to 70 °C. The materials were left on the hot plates until the solvent evaporated, and then they were thermostated at 80 °C for 48 h.

The preparation of the polyurethane nanocomposite involved the synthesis of prepolymer, followed by the addition of AHMP and graphene to the system using a solvent method. N,N-dimethylformamide was selected based on solubility studies of AHMP in various solvents such as dimethyl sulfoxide, tetrahydrofuran, and chloroform. During synthesis, the molar ratio of AHMP to BDO was 0.5 to 0.5, and the ratio between the NCO and OH groups was 1.05. At the same time, 60 mL of DMF was added to the beaker with AHMP, and stirring was initiated at 1000 rpm at 80 °C with a magnetic stirrer. The process of mixing AHMP in DMF was carried out for 6 h. Then, an appropriate amount of graphene was added to the AHMP and DMF system. The mixture was then placed in an ultrasonic bath to effectively disperse the graphene in solution. The ultrasonic process was carried out in “sweep” mode for 1 h. Subsequently, mechanical stirring was carried out at 800 rpm at 70 °C for 1 h.

The resulting dispersion was then introduced into the previously prepared prepolymer while increasing the stirring speed to 800–1000 rpm at 70 °C. Stirring continued for 20 to 30 min until a noticeable change in viscosity was observed. In the next step, a chain extender was added to the solution with AHMP and graphene, along with the catalyst. After these steps, the stirring speed was increased to 1500 rpm in order to obtain a homogeneous system. Subsequently, after 30 s, the entire reaction content was poured onto Teflon molds, which had been preheated to 70 °C. Then, after evaporation of the solvent, the molds with the cast materials were thermostated at 80 °C for 48 h. Preparation of materials differing in graphene content followed the same procedure.

**Figure 1:**
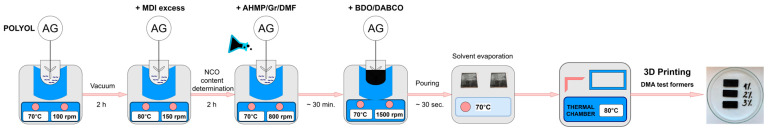
Polyurethane nanocomposites synthesis scheme.

A scheme for the synthesis of the nanocomposite is shown below in Figure 1.

Using the Tumaker NX Pro Pellets system, a filament was obtained, from which the samples in the form of DMA test formers were produced, meeting the requirements for the application of polyurethane nanocomposites with graphene addition in 3D printing. The printing process of the obtained nanocomposites was carried out using a 3D printer (Original Prusa i3 MK3S+ 3D Printer, Prague, Czech Republic). The processing parameters are presented in Table 1. The nozzle temperature refers to the temperature at the extruder nozzle during printing, while 1 Zone heating denotes a uniform barrel temperature maintained across the entire extruder using a single heating zone. The printing bed temperature is the setpoint of the heated platform, aiding in layer adhesion. The 1-layer extrusion and 2-layer extrusion values indicate the extrusion multipliers (in percentage) applied during the printing of the first and subsequent layers, respectively, which affect material flow and bonding. The printing speed, measured in mm/s, is the rate at which the print head moves during deposition. These parameters were optimized individually for each composite formulation to ensure printability and quality. Relative stability in the printing process parameters (Table 1), even with varying graphene contents, allowed for the creation of stable PU nanocomposites through 3D printing technology. This also creates new opportunities for their practical applications.

### 2.3. Method

Thermal studies were carried out using a NETZSCH TG 209F3 Thermogravimetric Analyzer, Selb, Germany (TGA) in the temperature range of 35–800 °C, at a heating rate of 10 °C/min, and under an inert N_2_ gas atmosphere; a TA Instruments Discovery DSC 250 Differential Scanning Calorimeter, Delaware, US (DSC) in the temperature range of −70–250 °C, at a heating rate of 10 °C/min, and under an inert N_2_ gas atmosphere; a TA Instruments RSA-G2 Rheological Analyzer, Delaware, US (dynamic mechanical analysis (DMA), equipped with a highly sensitive position sensor and linear motor with temperature-compensated rare-earth magnets) in the temperature range of −40–100 °C at a heating rate of 4 °C/min. Bar-shaped samples were analyzed: their dimensions were 10 × 2 × 1 mm, they were in tensile mode with linear geometry, and they had a force of 2 N and a strain of 0.01%. The structure of the obtained nanocomposites was analyzed using a FTIR spectrophotometer in ATR mode (IRTracer-100, Shimadzu, Kyoto, Japan) over a spectral range of 4000–500 cm^−1^, utilizing a nominal resolution of 4 cm^−1^, and comprised a total of 64 scans. The phase composition was analyzed by the X-ray diffraction method (XRD) using a Bragg–Brentano X’Pert Philips (Eindhoven, The Netherlands) diffractometer (40 kV, 30 mA, λ Cu Ka = 0.1542 nm). All samples were scanned from the 10 to 70° 2θ range.

For optical studies, a Keyence VHX-5000 Digital Microscope (Osaka, Japan) was used; images were taken at 200× magnification. Scanning Electron Microscopy (SEM) was performed on a FlexSEM 1000 II scanning electron microscope (Hitachi, Tokyo, Japan). The electron beam accelerating voltage was 10 kV. Each sample was coated with a thin double layer of gold to increase conductivity (using 108 Auto Sputter Coater, Cressington, UK). Hardness tests were also conducted using a Zwick/Roell hardness tester according to the PN-EN ISO 868: 2005 standard. The hardness measurements, expressed in Shore A degrees, represent the arithmetic mean of five individual measurements. Mechanical properties were analyzed using Universal Testing Systems Instron 68SC-5 (Norwood, MA, USA) with 5 kN grips at a crosshead speed of 50 mm/min according to ASTM D638 type 5 standards, Standard Test Method for Tensile Properties of Plastics, West Conshohocken, PA, USA, 2014.

## 3. Results and Discussion

### 3.1. TGA Studies

**Figure 2:**
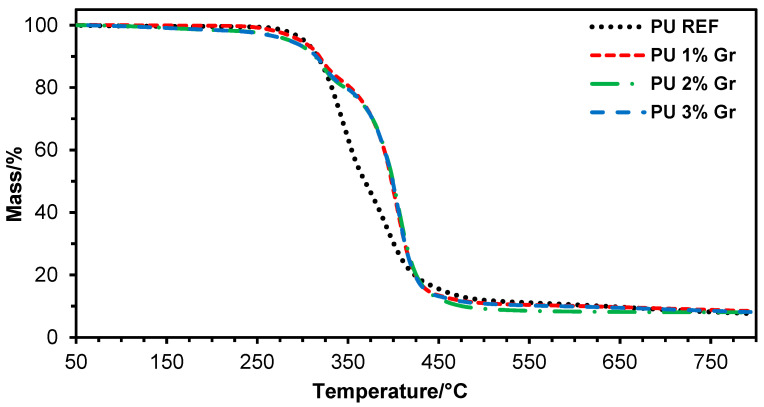
TGA curves for reference sample and samples with 1%, 2%, and 3% graphene (**--** PU REF, **--** PU 1% Gr, **--** PU 2% Gr, and **--** PU 3% Gr).

**Figure 3:**
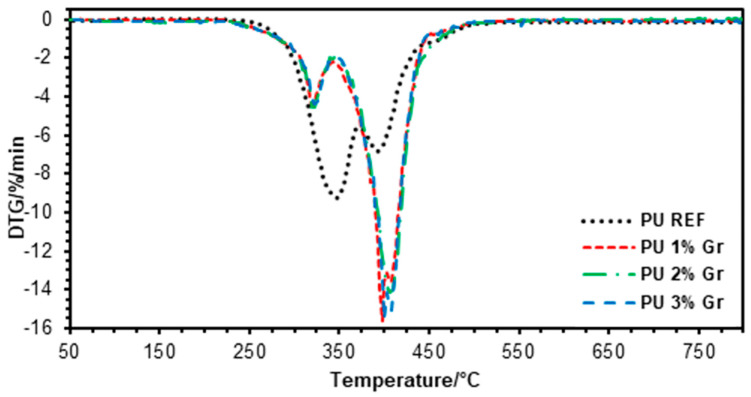
DTG (derivative thermogravimetric) curves for reference sample and samples with 1%, 2%, and 3% graphene (**--** PU REF, **--** PU 1% Gr, **--** PU 2% Gr, and **--** PU 3% Gr).

To characterize the thermal stability of the obtained polyurethane nanocomposites, tests were carried out using thermogravimetric analysis, which allows for the monitoring of changes in material mass as a function of temperature or time in a controlled atmosphere. The results are presented as temperature-dependent weight loss (Figure 2) and derivative weight loss (Figure 3).

The results of the thermogravimetric analysis (Figure 2 and Figure 3; Table 2) indicate a similar thermal stability of polyurethanes containing graphene compared to the material without a modifier (PU REF). This is particularly important in the initial phase of nanocomposite degradation. The material containing 1% graphene addition exhibits slightly higher thermal stability in the initial stage of degradation than nanocomposites with 2% and 3% of this additive. The T_d2%_ (272 °C) and T_d5%_ (298 °C) values for PU 1% Gr are higher than the analogous % mass loss parameters for PU 2% Gr and PU 3% Gr, which may indicate that a moderate concentration of graphene disrupts the structure of the obtained polyurethane to a lesser extent. Differential thermal analysis (DTA) revealed that all samples displayed a characteristic two-stage thermal degradation profile. The initial degradation stage corresponds to the disintegration of the main polymer chain, associated with the dissociation of urethane groups, followed by a second degradation phase attributed to the decomposition of soft polyester segments. For the subsequent stages of the maximum degradation rate, the highest temperature values were obtained for the material with 2% graphene, reaching, respectively, T_d2max_ = 398 °C and T_d3max_ = 406 °C. On this basis, it can be stated that a 2% graphene content in the sample may stabilize the material in this range as the recorded values of T_d2max_ = 347 °C and T_d3max_ = 393 °C for PU REF are significantly lower, which indicates that the presence of graphene improves the material’s resistance to thermal degradation in the later stages of decomposition. A 1–2% addition of graphene may provide optimal thermal properties of the nanocomposites in the medium temperature range, probably through a more uniform distribution of nanoparticles, which stabilizes the material in this phase of decomposition.

### 3.2. DSC Studies

**Figure 4:**
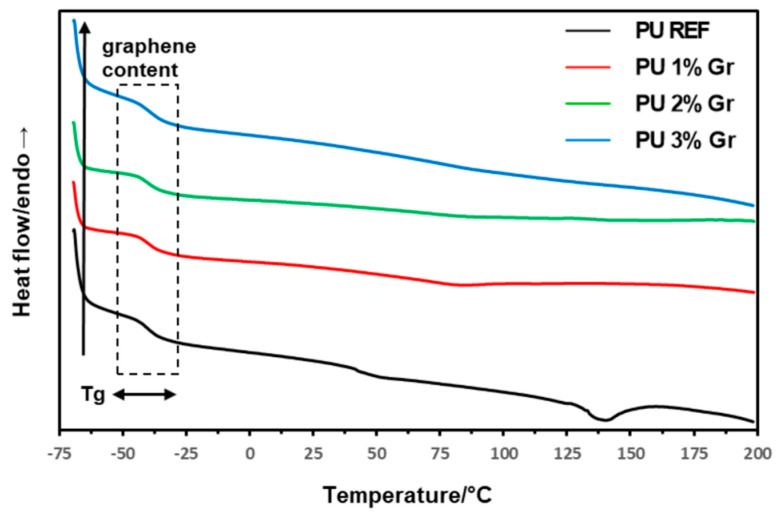
DSC 1st heating curves for reference sample and samples with 1%, 2%, and 3% graphene (**--** PU REF, **--** PU 1% Gr, **--** PU 2% Gr, and **--** PU 3% Gr).

**Figure 5:**
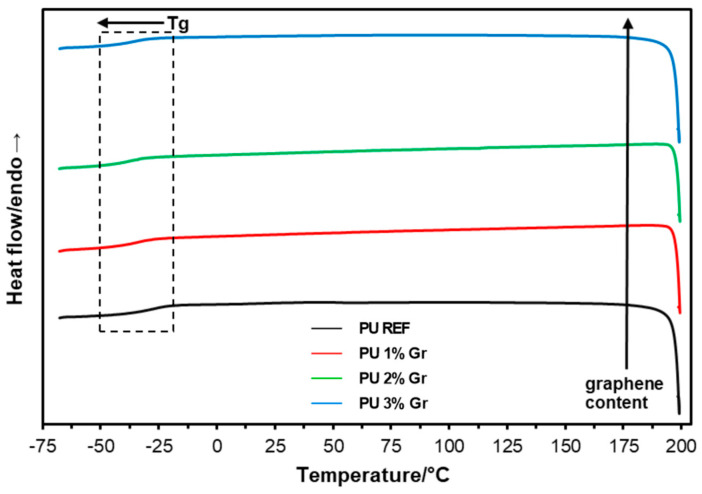
DSC cooling curves for reference sample and samples with 1%, 2%, and 3% graphene (**--** PU REF, **--** PU 1% Gr, **--** PU 2% Gr, and **--** PU 3% Gr).

**Figure 6:**
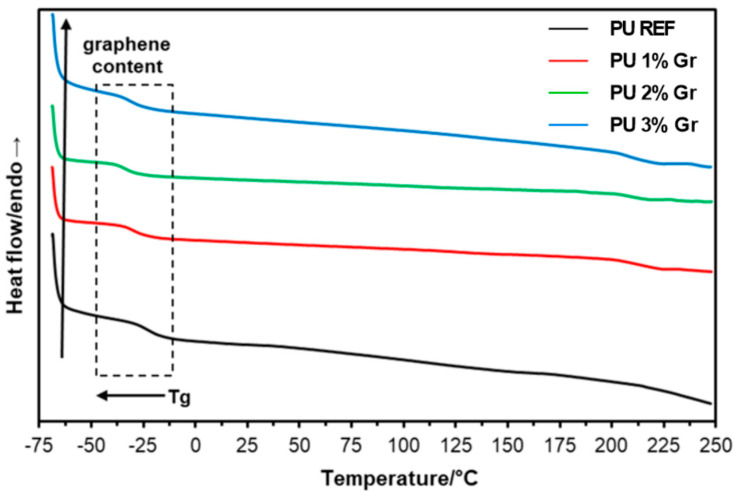
DSC 2nd heating curves for reference sample and samples with 1%, 2%, and 3% graphene (**--** PU REF, **--** PU 1% Gr, **--** PU 2% Gr, and **--** PU 3% Gr).

The phase transitions of the produced polyurethane materials were characterized using Differential Scanning Calorimetry (DSC). The obtained systems were analyzed by performing heating and cooling runs to determine key parameters such as the glass transition temperature (T_g_) (Table 3). The thermograms from the first and second heating runs and the cooling stage are presented in Figure 4, Figure 5 and Figure 6. The graphs also indicate the direction of changes with the increasing content of the nanofiller—graphene—and glass transition behavior.

The DSC curves in the analyzed temperature range indicate a more amorphous character of the obtained materials, both in the case of unmodified polyurethane (PU REF) and materials with graphene additive (PU 1–3% Gr). This may suggest a slight influence of the nanomodifier, which does not cause an increase in crystalline structures associated with the hard segments of polyurethanes. On the other hand, the presence of graphene undoubtedly affects changes in the glass transition temperature (T_g_) region of the materials. During the first heating cycle (Figure 4), the visible gradual transition process for all materials exhibited similar T_g_ temperature values equal to −40.0 °C (Table 3).

After removing the thermal history (heating the material at a controlled rate to its melting point, then continuing the analysis through controlled cooling and reheating cycles) of the materials, a decrease in the glass transition temperature (by approximately 7–11 °C) could be observed during cooling as the graphene content increased (Figure 5). This phenomenon may be related to an increase in the distance between the polyurethane chains caused by the addition of graphene nanofiller to the polymer matrix. A similar relationship was observed when the samples were reheated (Figure 6; Table 3), where the glass transition temperature was determined during the process. It also decreased from −28.5 °C recorded for PU REF to values 7–10 °C lower for graphene-modified materials.

### 3.3. DMA Studies

**Figure 7:**
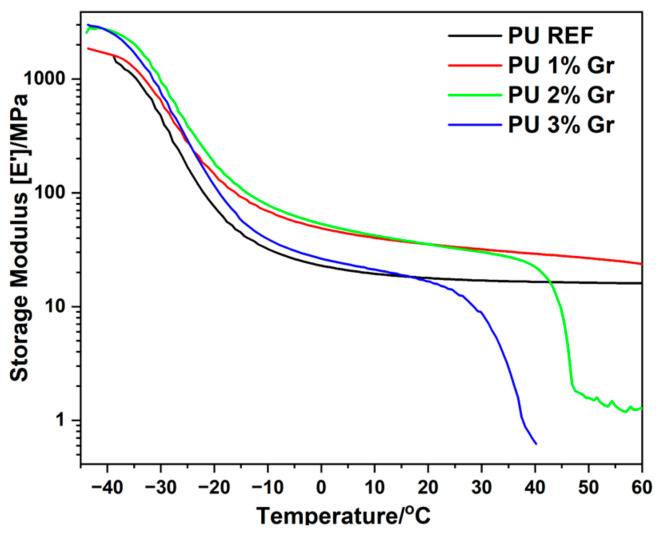
DMA curves of storage modulus E’ (MPa) versus temperature (°C) for all tested materials.

**Figure 8:**
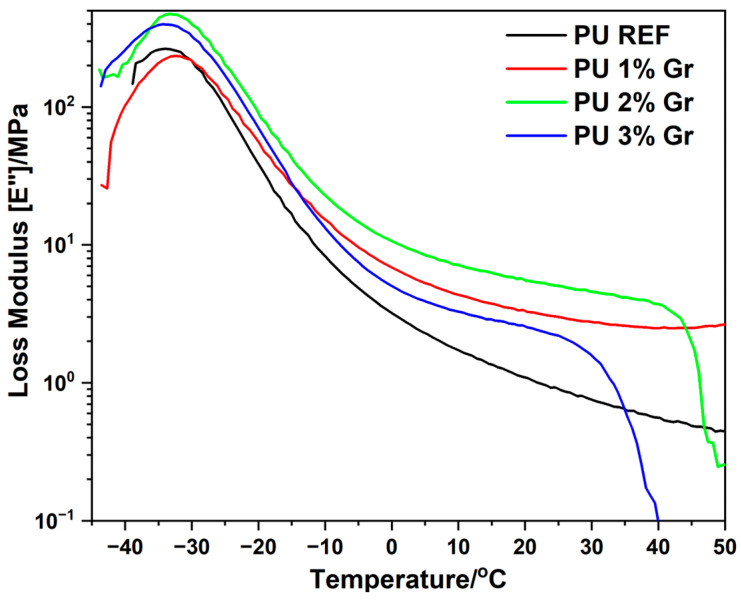
DMA curves of loss modulus E” (MPa) versus temperature (°C) for all tested materials.

**Figure 9:**
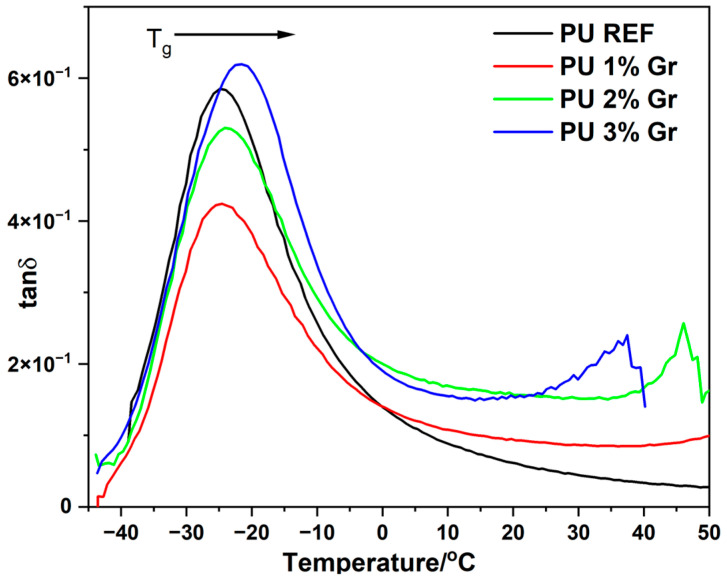
DMA curves of loss tangent (tan δ) versus temperature (°C) for all tested materials.

Dynamic mechanical analysis (DMA) was used to characterize the storage modulus (E′), loss modulus (E″), and damping factor (tan δ) of the polyurethane samples presented in Figure 7, Figure 8 and Figure 9. For the graphene-containing samples, the glass transition temperature (T_g_) was identified from the tan δ versus temperature curves. Although the T_g_ determined by DMA is slightly higher than that measured by DSC, both methods exhibit a comparable trend in their variations.

Polyurethane (PU REF) is characterized by a lower storage modulus (E′) across a wide range of analyzed temperatures, indicating less stiffness compared to polyurethane nanocomposites (Figure 7 and Table 4). As the graphene nanofiller content increases (1–2%), the E′ modulus rises, even above the glass transition temperature of the materials. A 3% additive of the modifier stabilizes the storage modulus (E′) to the level recorded for the unmodified material. At lower graphene concentrations (1–2%), optimal interaction likely enables effective stress transfer. However, at a 3% loading, if interfacial adhesion weakens—particularly above a certain temperature (e.g., 30 °C)—the polymer chains may begin to debond or slide over the graphene surfaces. This loss of interfacial integrity would significantly reduce the composite′s resistance to deformation.

Conversely, the glass transition temperatures (T_g_) determined from the maximum of the loss modulus (E″) (Figure 8 and Table 4) are observed in a similar temperature range of −32 to −34 °C. As the graphene content increased, the tan δ values experienced an elevation while the peak of tan δ diminished, indicating enhanced mobility of the molecular chains. The value of this parameter was also determined from the maximum of the loss tangent (Figure 9 and Table 4), where T_g_ values are around −21 °C to −24 °C. This indicates that the addition of graphene does not significantly affect the change in glass transition temperature (T_g_), suggesting no plasticizing effect of the nanomodifier.

### 3.4. FTIR Spectroscopic Studies

**Figure 10:**
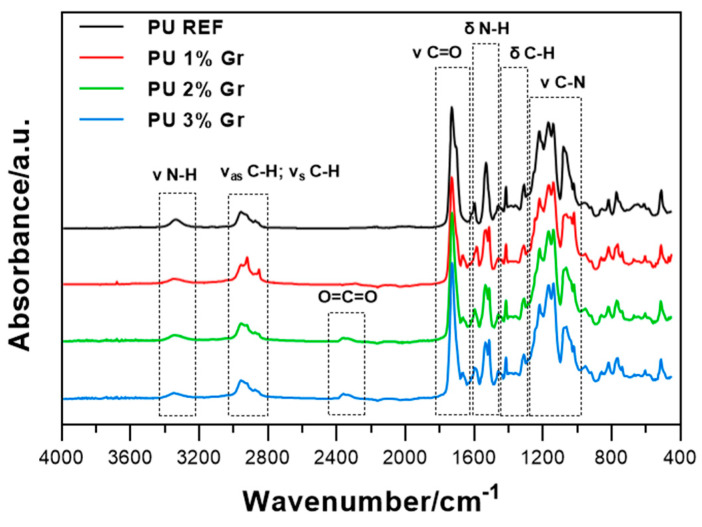
FTIR spectra for PU REF and PU (1–3%) Gr samples.

FTIR spectroscopy, with the results presented in Figure 10, was carried out to identify the characteristic chemical groups present in the synthesized polyurethanes based on the absorption band positions in the FTIR spectrum. Polyurethane systems typically exhibit a characteristic two-phase structure where urethane and urea bonds can form hydrogen bonds, in this case with ureidopyrimidone units, reinforcing the hydrogen bond network.

For each sample the characteristic vibrations of urethane and urea groups were observed around 3340 cm^−1^, attributable to the N-H stretching vibrations. In this region, the characteristic N–H stretching peak at 3270–3400 cm^−1^ appeared as a non-separate peak. The symmetric and asymmetric stretching vibrations of -CH_2_- appeared at 2956 cm^−1^ and 2870 cm^−1^, respectively. Peaks at approximately 1730 cm^−1^ corresponded to the C=O stretching vibration of urethane, indicating the presence of ester and urethane linkages within the polyurethane structure. The absorption band around 1530 cm^−1^ corresponded to the out-of-plane bending of the NH bond and the CN stretching vibrations. The absence of a characteristic absorption peak for -NCO ~2270 cm^−1^ indicated the completion of reactions in all samples. An absorption peak around 2360 cm^−1^ with a characteristic two-stage step was observed for PU 2% Gr and PU 3% Gr samples, originating from carbon dioxide.

The reference material (without graphene) displays the highest intensity within the band around 1730 cm^−1^, which is attributed to carbonyl groups (C=O). When comparing polyurethane nanocomposites to the reference material, a decrease in the intensity of the carbonyl band (1730 cm^−1^) is observed. This suggests a potential interaction between graphene and the polyurethane matrix, affecting the carbonyl bond structure. Moreover, peaks near 1660 cm^−1^ visible for samples PU 1–3% Gr are attributed to the C=O stretching vibrations of ureidopyrimidone, confirming the incorporation of the UPy group into the polymer matrix. PU REF devoid of AHMP and thus UPy groups displayed an absence of an absorption peak at 1660 cm^−1^. Furthermore, changes in the intensity of the band at 3330 cm^−1^ in the presence of graphene may indicate the additive’s influence on the cross-linking and orientation of amide groups within the matrix. An increase in the graphene content (1–2%) leads to noticeable changes in the intensity and shape of the spectra in the 1400–1800 cm^−1^ region. This may suggest that a greater amount of graphene influences the microstructure of the polyurethane, likely reinforcing it. At higher graphene concentrations (2% and 3%), the changes in band intensity are more pronounced than at 1% graphene addition, which can be attributed to the restriction of polymer segment mobility and mutual interactions between graphene and the polymer. In summary, the addition of AHMP and graphene to the polyurethane matrix affects its chemical structure, which is evident in the FTIR spectra as changes in band intensity and position. Increasing the graphene content leads to structural modifications that can be translated into the physicochemical properties of the composites, such as thermal stability, flexibility, and tensile strength. The mutual interaction between the polyurethane matrix and graphene can lead to the reinforcement of the polyurethane structure. An increased number of functional groups influencing vibrational translations or material stiffening may enhance the potential application of these composites in areas requiring higher mechanical stability.

### 3.5. XRD Studies

**Figure 11:**
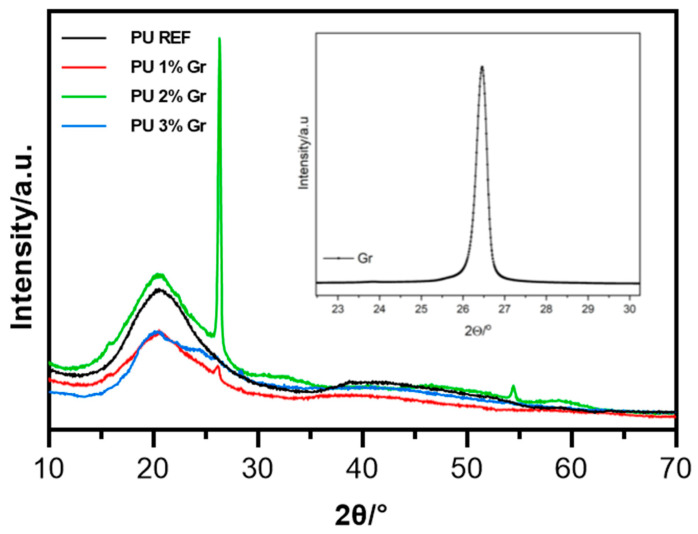
X-ray diffraction patterns (XRD) for PU REF and PU (1–3%) Gr samples.

The XRD patterns for polyurethane samples revealed a prominent peak at approximately 2θ = 20°, along with variations in peak intensities and slight shifts in their positions, as presented in Figure 11. The diffraction peaks observed near 20° have been attributed to the amorphous structure of the materials; the broad peak indicates the predominance of an amorphous polyurethane matrix, while the presence of sharper peaks suggests the existence of crystalline regions. The differences in peak intensities and minor shifts may reflect variations in the degree of crystallinity or the distribution of crystalline domains among the different samples, potentially linked to the respective amounts of added components. Samples incorporating graphene exhibited decreased peak intensity compared to those without nanoparticle additions. The peak at approximately 2θ = 26° is indicative of graphene, suggesting its presence and potential exfoliation within these samples. The variability in peak intensities at this angle may correlate with differences in graphene dispersion or its interaction with the polymer matrix. The sharp peak at 26° in the 2% Gr sample likely indicates partial crystallinity due to graphene restacking or aggregation, possibly driven by suboptimal dispersion or polymer–graphene interactions at that loading. At 1%, the graphene is too dispersed to exhibit crystallinity; at 3%, restacking may be suppressed by better matrix interaction or random stacking disorder. Additionally, samples containing UPy groups displayed numerous sharp peaks, which can be attributed to the formation of large crystalline assemblies resulting from extensive hydrogen bonding. This observation aligns with the known propensity of UPy to create substantial crystalline stacks during assembly and to facilitate microphase separation, leading to the development of supramolecular polymer networks. The XRD analysis effectively illustrates the influence of graphene and other additives on the morphology of the polyurethane matrix. Such alterations are critical for optimizing material properties for specific applications, including mechanical strength, electrical conductivity, and thermal stability.

### 3.6. Self-Healing Properties Studies

The relatively low glass transition temperature (Tg) of amorphous polymers is a critical parameter as it may enable the manifestation of self-healing properties near room temperature (RT). The self-healing mechanism is facilitated by the thermal reversibility of multiple hydrogen bonds, which exhibit particularly strong interactions at elevated temperatures exceeding 80 °C. During the heating phase, the cross-linking network among the polymer molecules is disrupted due to the temporary cleavage of multiple hydrogen bonds, resulting in a notable increase in intermolecular mobility. As the samples cool, the multiple hydrogen bonds recombine due to their inherent reversibility. Consequently, the scratch on the film progressively heals through this reversible dynamic process [28].

**Figure 12:**
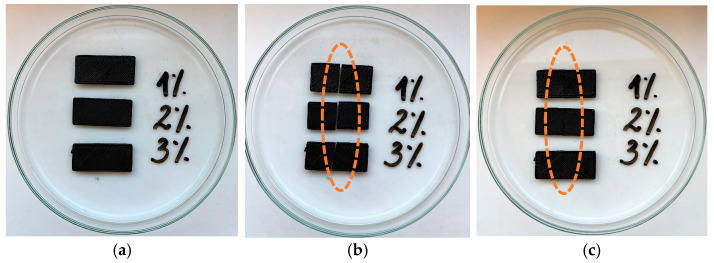
PU specimens with 1–3% graphene content. (**a**) Materials before cutting, (**b**) materials after cutting, and (**c**) materials after being in an oven (at 80 °C for an hour).

The research procedure for investigating the self-healing properties of 3D-printed post-processed materials (shaped specimens) was the same as for raw materials. All specimens used were 30 mm long and 1 mm wide (Figure 12a). After cutting the materials (Figure 12b), all fragments could be continuously rejoined at the cutting site. All materials exhibited improved self-healing properties; however, the most effective temperature for their activation was 80 °C. It was observed that after removing the materials from the oven, set at 80 °C, the cut sites were barely visible (Figure 12c).

**Figure 13:**
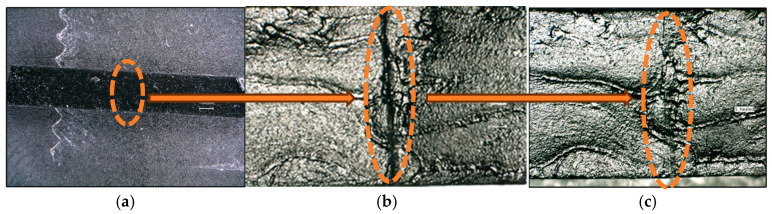
PU specimens with 2% graphene content. (**a**) Material before cutting, (**b**) material after cutting, and (**c**) material after self-healing process.

Moreover, for the self-healing assessment, dumbbell-shaped samples were deliberately scratched with a scalpel. Subsequently, the two segments were placed in contact and maintained at 80 °C for an hour, followed by observation at room temperature using a digital optical microscope. Microscopic observations were conducted in three stages: before mechanical damage, after mechanical damage (a cut along the material), and after the regeneration process. This allowed for the detection of structural (morphological) changes occurring in the materials at each stage of the study. After the self-healing process (Figure 13c), the line of the previous damage (Figure 13b) was closed. It was observed that the presence of UPy groups and graphene promoted the closing of damage and improved structural organization in the repair area, indicating the effectiveness of the material regeneration process.

### 3.7. SEM Studies

**Figure 14:**
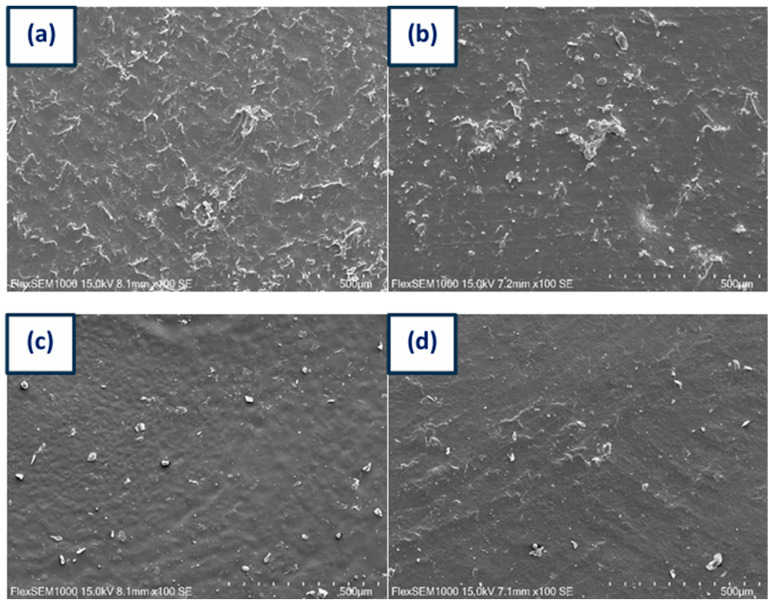
SEM images of PU specimens. (**a**) PU REF, (**b**) PU 1% Gr, (**c**) PU 2% Gr, and (**d**) PU 3% Gr.

The structural characteristics of polyurethanes were examined through scanning electron microscopy analysis, and the results are presented in Figure 14. Image (a) depicts pure polyurethane devoid of any additives. The surface appears relatively smooth, exhibiting minor roughness and texture that is characteristic of neat polyurethane. Image (b) illustrates the polyurethane incorporating 1% graphene. The surface exhibits enhanced roughness and texture, with visible clusters or agglomerates confirming graphene presence, which appears to be uniformly distributed throughout the matrix, contributing to the increased surface roughness. Image (c) represents the polyurethane containing 2% graphene, with more pronounced surface roughness compared to image (b), suggesting a higher concentration of graphene particles. The presence of visible clusters indicates that graphene is more prominent and less uniformly dispersed in this formulation. Image (d) corresponds to the polyurethane with the highest graphene concentration, revealing significant surface roughness and a greater number of clusters relative to images (b) and (c). The elevated graphene content resulted in more substantial agglomeration, potentially impacting the material properties by leading to more heterogeneous regions.

### 3.8. Mechanical Properties

**Figure 15:**
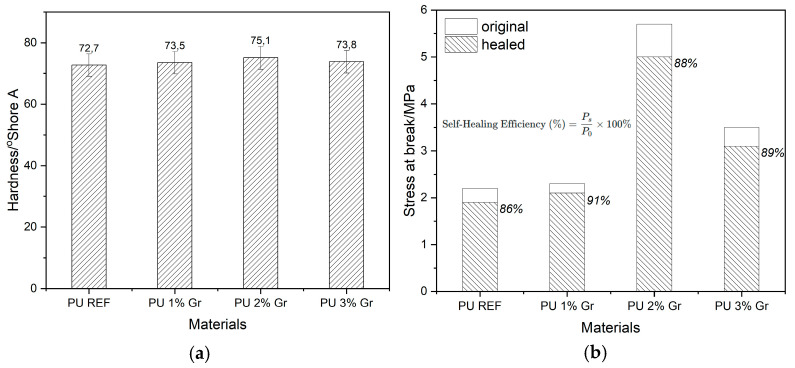
(**a**) Comparison of hardness (°Shore A) for all tested materials. (**b**) Stress at break (MPa) for original vs. healed materials. Calculation of self-healing efficiency based on mechanical properties [P_s_ = tensile strength of self-healed material; P_0_ = tensile strength of undamaged (original) material].

Hardness testing is a fundamental method used to assess the mechanical properties of materials like, for example, elastomers. In this hardness test, all obtained materials were analyzed, and a bar chart (Figure 15a) was prepared to illustrate the average Shore hardness value for each material. The unmodified material showed a hardness of approximately 73° Shore A. In contrast, the modified polyurethanes exhibited similar, slightly higher values for this parameter. The material containing a 2% graphene additive achieved the highest value, reaching 75° Shore A. Based on the results it can be concluded that the optimal graphene additive concentration for achieving the highest hardness without weakening the material’s structure is at the aforementioned 2% level.

The mechanical analysis (Figure 15b) reveals that the incorporation of AHMP and graphene significantly influences both the mechanical properties and the self-healing capabilities of PU materials. The optimal graphene content is 2% by weight, which maximizes the initial tensile strength of PU composites. A higher concentration (3%) appears to lead to a decrease in the original mechanical strength, possibly due to graphene aggregation. All graphene-reinforced PU composites exhibit high self-healing efficiencies (88–91%), slightly better than the neat PU (86%). This suggests that graphene does not negatively impact the intrinsic healing mechanism of the PU matrix at these concentrations, and in some cases, might even enhance it. PU with 2% graphene stands out as it achieves the highest initial mechanical strength while retaining excellent self-healing efficiency (88%). This combination makes it a promising candidate for applications requiring robust materials with intrinsic repair capabilities. The ability of the 2% Gr composite to recover a healed stress at break of over 5 MPa, which is higher than the original strength of the neat PU, highlights the synergistic benefits of graphene reinforcement and self-healing properties. The results are similar to other observations described in [31], where graphene/dimethylglyoxime–urethane polyurethan composites were synthesized. The mechanical strength of the self-healing materials was approximately 6 MPa, while the self-healing efficiency reached 92% after 96 h at room temperature.

## 4. Conclusions

This study shown that adding graphene to polyurethanes affects their thermal and mechanical properties, as well as their self-healing abilities. The results indicate varying effects depending on the graphene concentration, which allows the material’s parameters to be tailored to specific applications. Additionally, it has been confirmed that processing graphene-enriched polyurethanes using 3D printing technology is feasible. However, this requires careful selection of the printing system, precise control of parameters, and preliminary preparation of the material. Analyses indicate that a 2% graphene concentration in the polyurethane matrix is the most beneficial, where the mechanical strength has reached 5 MPa and the self-healing efficiency has reached 88%. A cut in the PU nanocomposite containing 2% graphene healed completely after heating at 80 °C for an hour, indicating a rapid recovery time. This concentration provides a balance between improved mechanical properties and thermal stability. Graphene at this concentration stabilizes the material at medium temperatures and enhances its stiffness. Of course, the final choice of graphene concentration depends on many factors and the specific application. Undoubtedly, in applications requiring high mechanical and thermal stability, it is recommended to use graphene at this level. Further research could investigate the mechanisms underlying the observed optimal graphene content for mechanical strength and the specific role of graphene in the self-healing process of these PU nanocomposites.

## Figures and Tables

**Table 1:** Processing parameters for 3D-printed PU materials with 1–3% graphene content.

Sample	Nozzle	1-Zone Heating	Printing Bed	1-Layer Extrusion	2-Layer Extrusion	Printing Speed
	°C	°C	°C	%	%	mm/s
PU 1% Gr	220	150	60	700	600	40
PU 2% Gr	215	150	60	450	450	40
PU 3% Gr	220	150	60	600	450	40

**Table 2:** Compilation of characteristic TGA and DTG curve values for PU materials.

Sample	T_d2%_	T_d5%_	T_d10%_	T_d2max_	T_d3max_
	°C	°C	°C	°C	°C
PU REF	283	302	315	347	393
PU 1% Gr	272	298	317	386	397
PU 2% Gr	237	287	312	398	406
PU 3% Gr	233	286	313	391	400

T_d2%_, T_d5%_, and T_d10%_—temperatures at 2%, 5%, and 10% weight loss; T_d2max_ and T_d3max_—peak maximum temperatures of DTG derivative.

**Table 3:** Glass transition temperatures (from DSC curves).

Sample	1st Heating	Cooling	2nd Heating
		Tg/°C	
PU REF	−39.9	−22.3	−28.5
PU 1% Gr	−39.9	−29.1	−35.1
PU 2% Gr	−40.0	−33.4	−38.4
PU 3% Gr	−39.2	−33.4	−36.9

**Table 4:** Glass transition temperatures (from DMA curves).

Sample	E′(0 °C)	E″_max_	Tan δ
	MPa	°C	°C
PU REF	22.9	−34.1	−24.6
PU 1% Gr	47.6	−32.2	−24.3
PU 2% Gr	53.3	−32.9	−23.6
PU 3% Gr	26.3	−33.7	−21.4

## Data Availability

Data are contained within the article.

## Abbreviations

The following abbreviations are used in this manuscript:

PU	Polyurethane
TPU	Thermoplastic polyurethane
MDI	4,4′-diphenylmethane diisocyanate
AHMP	2-amino-4-hydroxy-6-methylpyrimidine
UPy	Ureido-pyrimidinone
Gr	Graphene
GO	Graphene oxide
BDO	1,4-butanediol
DMF	N,N-dimethylformamide
DMA	Dynamic mechanical analysis
DTA	Derivative thermogravimetric
TGA	Thermogravimetric analyzer
DSC	Differential scanning calorimeter
FTIR	Fourier transform infrared spectroscopy
XRD	X-ray diffraction
SEM	Scanning electron microscopy
REF	Reference sample
Td	The temperature at the initial decomposition
Tg	Glass transition temperature
DTA	Differential thermal analysis
E′	Storage modulus
E″	Loss modulus
tanδ	Loss tangent
